# Supplementary material for: The interactome of intact mitochondria by cross-linking mass spectrometry provides evidence for coexisting respiratory supercomplexes
Source: Mol Cell Proteomics. 2017 Dec 8;17(2):216–32. doi: 10.1074/mcp.RA117.000470 (PMC5795388; doi:10.1074/mcp.RA117.000470)
Supplement: Supplemental Data [file supp_RA117.000470_133922_0_supp_23978_fzffwf.zip › spectra_annotation/mito_DR_spectra_annotation/326-1-6-1-3-1.pdf]

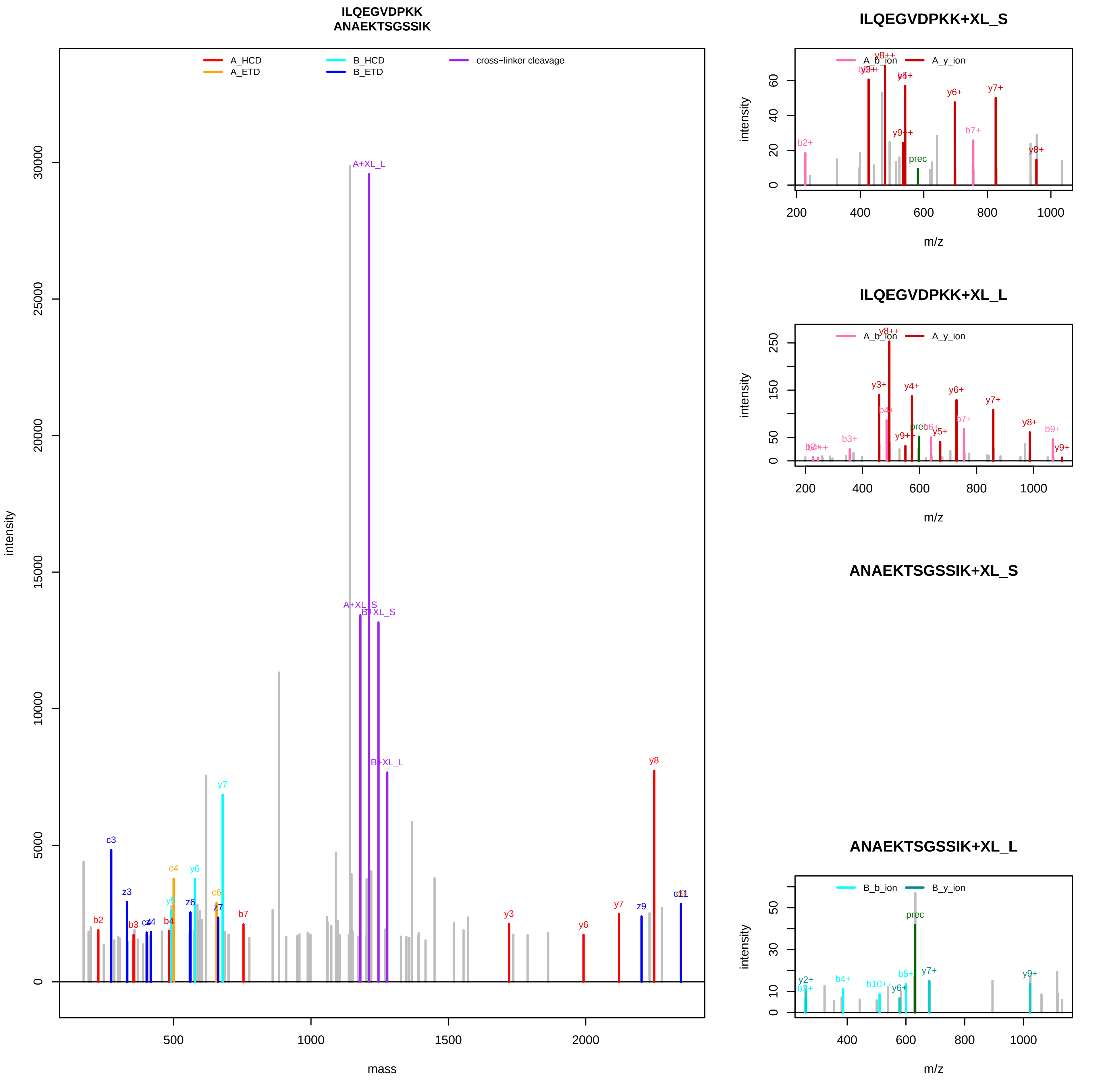
ILQEGVDPKK
ANAEKTSGSSIK
A_HCD
A_ETD
B_HCD
B_ETD
cross−linker cleavage
A+XL_L
A+XL_S
B+XL_S
B+XL_L
c3
z3
b2
b3
c4
z4
b4
c4
y5
y6
z6
c6
z7
y7
b7
y3
y6
y7
z9
y8
c11
intensity
mass
0
5000
10000
15000
20000
25000
30000
500
1000
1500
2000
ILQEGVDPKK+XL_S
A_b_ion
A_y_ion
b2+
b3+
y3+
y8++
y4+
y9++
prec
y6+
b7+
y7+
y8+
intensity
m/z
ILQEGVDPKK+XL_L
A_b_ion
A_y_ion
y8++
y3+
y4+
b4+
y6+
y7+
b7+
prec
b6+
y5+
y9++
b3+
y8+
b9+
y9+
intensity
m/z
ANAEKTSGSSIK+XL_S
ANAEKTSGSSIK+XL_L
B_b_ion
B_y_ion
prec
y2+
b3+
b4+
b10++
b5+
y6+
y7+
y9+
intensity
m/z
